# Shape, colour plasticity, and habitat use indicate morph-specific camouflage strategies in a marine shrimp

**DOI:** 10.1186/s12862-016-0796-8

**Published:** 2016-10-18

**Authors:** Rafael C. Duarte, Martin Stevens, Augusto A. V. Flores

**Affiliations:** 1 Centro de Biologia Marinha, Universidade de São Paulo, São Sebastião, Brazil; 2 Programa de Pós-Graduação em Biologia Comparada, Faculdade de Filosofia, Ciências e Letras de Ribeirão Preto, Universidade de São Paulo, Ribeirão Preto, Brazil; 3 Centre for Ecology and Conservation, College of Life and Environmental Sciences, University of Exeter, Penryn Campus, Penryn, Cornwall TR10 9FE UK

**Keywords:** Camouflage strategy, Caridean shrimp, Polymorphism, Geometric morphometrics, Colour change, Habitat use, Life-styles

## Abstract

**Background:**

Colour and shape polymorphisms are important features of many species and may allow individuals to exploit a wider array of habitats, including through behavioural differences among morphs. In addition, differences among individuals in behaviour and morphology may reflect different strategies, for example utilising different approaches to camouflage. *Hippolyte obliquimanus* is a small shrimp species inhabiting different shallow-water vegetated habitats. Populations comprise two main morphs: homogeneous shrimp of variable colour (H) and transparent individuals with coloured stripes (ST). These morphs follow different distribution patterns between their main algal habitats; the brown weed *Sargassum furcatum* and the pink-red weed *Galaxaura marginata*. In this study, we first investigated morph-specific colour change and habitat selection, as mechanisms underlying camouflage and spatial distribution patterns in nature. Then, we examined habitat fidelity, mobility, and morphological traits, further indicating patterns of habitat use.

**Results:**

H shrimp are capable of changing colour in just a few days towards their algal background, achieving better concealment in the more marginal, and less preferred, red weed habitat. Furthermore, laboratory trials showed that habitat fidelity is higher for H shrimp, whereas swimming activity is higher for the ST morph, aligned to morphological evidence indicating these two morphs comprise a more benthic (H) and a more pelagic (ST) life-style, respectively.

**Conclusions:**

Results suggest that H shrimp utilise a camouflage strategy specialised to a limited number of backgrounds at any one time, whereas ST individuals comprise a phenotype with more generalist camouflage (transparency) linked to a more generalist background utilisation. The coexistence within a population of distinct morphotypes with apparently alternative strategies of habitat use and camouflage may reflect differential responses to substantial seasonal changes in macroalgal cover. Our findings also demonstrate how colour change, behaviour, morphology, and background use all interact in achieving camouflage.

**Electronic supplementary material:**

The online version of this article (doi:10.1186/s12862-016-0796-8) contains supplementary material, which is available to authorized users.

## Background

Polymorphism is a common trait in many animal taxa [1, 2] and has been a subject of numerous empirical studies testing several evolutionary theories and hypotheses (e.g. [3–5]). Aside from facilitating the exploitation of a wider array of habitats [6–9], polymorphism may also involve a segregation of behavioural traits among morphs, such as related to differences in mating tactics [10, 11] or habitat use [12, 13]. Morph-specific morphological and behavioural traits can allow individuals to more efficiently gather resources and exploit different niches through the diversification and specialisation of life-history strategies [6, 14, 15]. Identifying the selective forces responsible for the origins and maintenance of morphs, and unravelling their relative advantages, are important tasks in order to predict population dynamics in varying environments and for understanding evolutionary and developmental strategies [2, 16, 17].

One of the most longstanding areas where colour and shape polymorphisms have been studied in nature relates to camouflage [4, 8, 18–20]. Habitat-specific camouflage of colour morphs may be obtained via a number of mechanisms, whereby behavioural and morphological traits of individuals can interact with environmental characteristics to reduce their relative risk of predation [21–23]. For instance, individual appearance for camouflage can be either attained through genetic polymorphism [24, 25], or through colour change and phenotypic plasticity [26–28]. In addition to changes in appearance, camouflage can also be driven by the behavioural preferences of individuals to rest on backgrounds that provide enhanced camouflage [23, 29, 30]. Evidence of morph-specific behavioural preferences for substrate types has been observed in a variety of taxa, including moths [31], grasshoppers [32] and crabs [7], and we would expect this to be common if morphs have evolved under selection for camouflage against different substrates. Therefore, camouflage in polymorphic species should be driven by both colour change in line with the predominant visual background, and behavioural preferences for individuals to rest on backgrounds that they match.

The degree to which different morphs can exploit alternative (micro) habitats should depend on how effectively individuals can conceal themselves against the background. Therefore, for species living in heterogeneous substrates, different morphs may be effectively concealed in microhabitats with different background colour patterns within the same general environment [17, 33, 34]. In this case, predation by visual consumers may drive disruptive selection leading to individuals specializing in each of the available backgrounds [35, 36], and/or the ability of some individuals to change colour depending on the patch type they live on [26, 37, 38]. On the other hand, a more generalist fixed strategy may be favoured when optimal colouration is achieved by a compromise in the degree of crypsis obtained in different microhabitats while matching no background very closely [33, 39], or through camouflage types that are less restricted to one background type alone (e.g. transparency).

Differential coloration and camouflage strategies may evolve together with both morphological and behavioural traits in polymorphic species [21, 32]. For example, colour patterns in Midas cichlid fish are correlated to both body shape and life-style, with golden deeper bodied fish mostly associated to the benthic habitat, and dark slender individuals exhibiting a more limnetic life-style [15]. Also, Dalmatian wall lizards comprise three different colour morphs, with different body and relative head size, which relate to morph-specific trophic niches and cross-habitat distributions [40]. Theory also predicts that morphs with a specialist camouflage strategy would concentrate in habitat patches where concealment is most efficient, increasing substrate fidelity and lowering predation risk [32, 41]. Active preference for these patches may lead to exceptionally high population densities, only constrained by habitat carrying capacity [42], favouring high intra-specific competition, with some individuals being displaced to marginal habitat patches [43]. Alternatively, for individuals with a generalist strategy, in which camouflage is less constrained to a limited number of backgrounds, selection may favour a more opportunistic life-style with individuals possessing differential morphology and behaviour [17, 44]. A generalist life-style with lower habitat fidelity and increased mobility may allow individuals to reduce competitive interactions and facilitate more efficient resource exploitation and mate searching [45, 46]. Strong specialization, coupled to habitat fidelity, and high mobility associated to a more opportunistic use of resources, can be found in different morphs within populations, and their coexistence is apparently mediated by environmental conditions dictating relative fitness of individuals at different frequencies [47, 48].

**Fig. 1:**
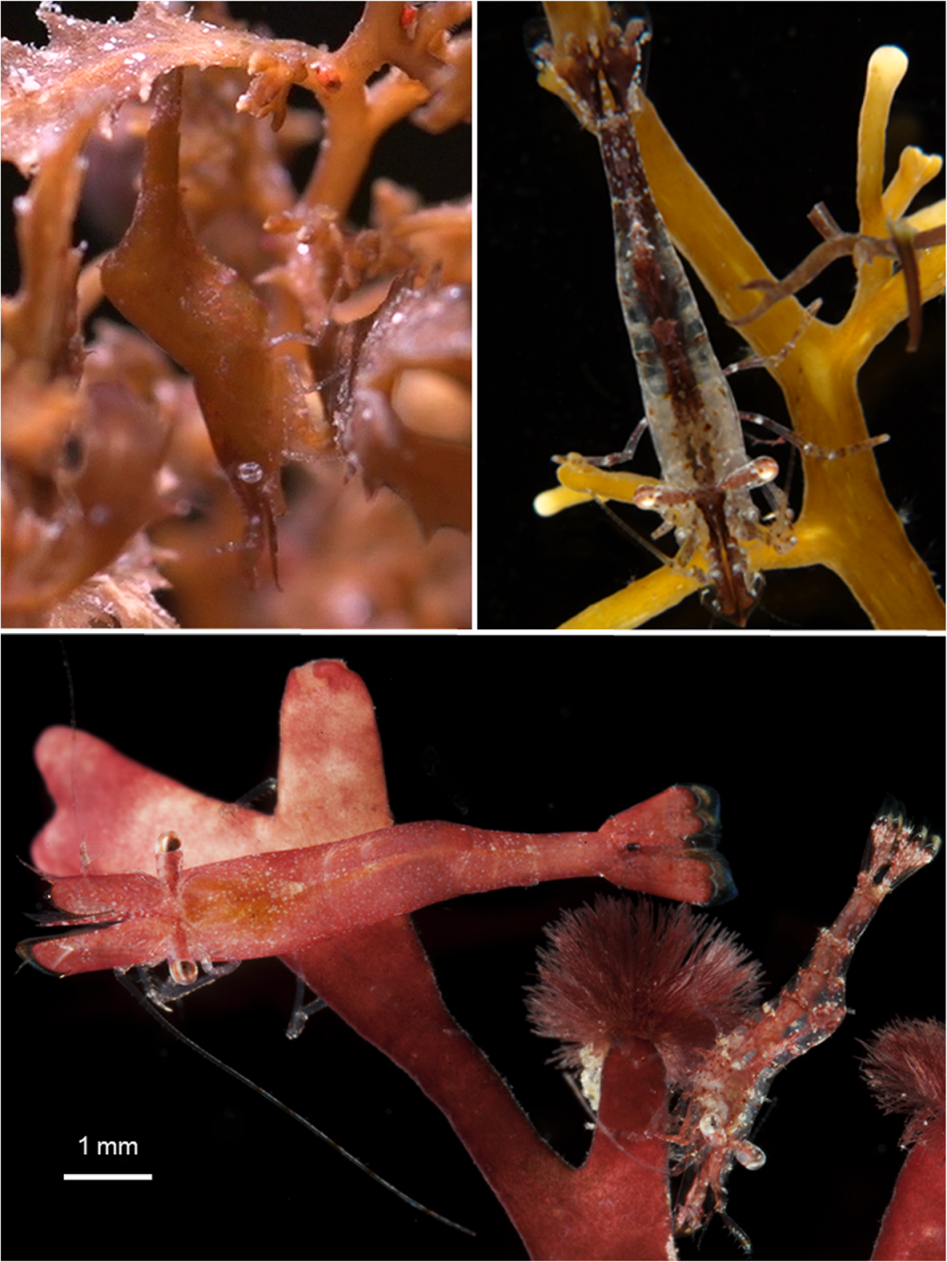
*Hippolyte obliquimanus* colour morphs. Homogeneous (H) individuals characterized by a greenish-brown (H_GB_: *top-left* shrimp) or pink (H_P_: *bottom-left* shrimp) coloration attaining a good colour match in the brown algae *Sargassum furcatum* and the red-pink weed *Galaxaura marginata*, respectively. Striped translucent (ST) individuals bearing longitudinal colour bands (*top-right* and *bottom-right* shrimp), showing a general resemblance to both algae

The shrimp *Hippolyte obliquimanus* is a small gonochoric and polymorphic species [49], very abundant in algal meadows composed of *Sargassum furcatum* (hereafter *Sargassum*) and *Galaxaura marginata* (from now on *Galaxaura*), the dominant algal species of shallow rocky substrates in the northern coast of São Paulo State, Brazil [9]. Shrimp populations comprise distinct morphs, all belonging to the same species [50], which can be classified as (*i*) homogeneous individuals (H) with different coloration, most being greenish-brown (H_GB_) or pink (H_P_), and (*ii*) striped translucent shrimp (ST), with either longitudinal or transversal colour bands (Fig. 1). Homogeneous shrimp are visually well concealed in both the brown alga *Sargassum* (H_GB_) and the reddish-pink seaweed *Galaxaura* (H_P_), while ST individuals, although found in these same habitats, exhibit less background-specific coloration via the use of transparency (Fig. 1).

The natural distribution of *H. obliquimanus* individuals between algal habitats is clearly morph-specific [9]. H individuals tend to occupy colour-matching substrates, i.e. greenish-brown shrimp are more abundant in *Sargassum*, while pink individuals in *Galaxaura*, and ST shrimp are equally distributed between these macroalgae [9]. While over a period of days and weeks H shrimp may be able to change colour to different substrate types (see below), at any one time they should be restricted to one matching background type alone, and hence we consider them background specialists (but note that over time they may be considered generalists). Mismatching shrimp, i.e. H_GB_ in *Galaxaura* or H_P_ in *Sargassum*, are very probably individuals that arrived from a different habitat and had not yet adjusted to local background. In contrast, ST individuals may adopt more generalist background choice behaviour and a camouflage type (transparency) that allows concealment to a range of substrate types. Sex proportions are also different between morphs, with H shrimp being chiefly females and ST mostly males, suggesting that selection for sex-specific traits may be also important in explaining the maintenance of polymorphism in this species [9]. Morph-specific habitat and sex distribution may indicate the existence of behavioural differences between morphotypes [32, 51], possibly related to contrasting strategies of habitat use. In the case of *H. obliquimanus*, cryptic behaviour is expected to be selected in H shrimp, with individuals remaining on colour-matching backgrounds, and a more general life-style is anticipated for transparent ST individuals, which would move more frequently among different substrate types.

In this study, we used a combination of laboratory manipulative experiments, supported by geometric morphometric analyses, to test the hypothesis that colour morphs of *H. obliquimanus* differ in specific behavioural traits and morphology related to strategies of camouflage and habitat use (namely *Sargassum* and *Galaxaura* canopy). We first examined two potential mechanisms by which individuals can enhance crypsis: habitat selection and colour change. We undertook experiments of behavioural habitat selection to test whether morphs actively select the background-matching macroalgal habitat where concealment is more effective. Then, we performed a colour change experiment to investigate if the capacity of colour change differs between morphs and habitats. Because carapace shape can be a proxy for life-style and habitat use in caridean shrimps, with stout forms being an indicative of benthic life-style and more streamlined shapes of a more pelagic behaviour [52, 53], we used geometric morphometric analyses and carried out experiments of habitat use to verify whether morphological evidence correlates with behavioural patterns. Together, the results of this study evidenced a link among coloration, morphological, and behavioural traits, illustrating how polymorphism can be advantageous to individuals achieve different camouflage strategies when living in a heterogeneous habitat.

## Methods

### General procedures

Samples of the macroalgae *Sargassum* and *Galaxaura* were collected during the summer and autumn of 2011, 2013 and 2015 by skin diving at rocky bottoms in different sites along the São Sebastião Channel (23°49′38″S; 45°25′16″W; São Sebastião, SP, Brazil). Individuals of *H. obliquimanus* were sorted out from the macroalgae (as in [9]), visually classified as H_GB_, H_P_ or ST, and used in laboratory experiments to compare morph-specific algal preferences, colour change capacities and behaviour. We validated this visual classification by running a discriminant function analysis (DFA), using the ‘lda’ function from the package MASS in R [54], on random samples of individuals initially classified as H_GB_ and H_P_ (*n* = 10), to which colour reflectance values in image RGB colour channels were measured (as described below in ‘Colour change and camouflage’). DFA scores for these morphs were discrete and non-overlapping (DFA scores: −5.54 < H_GB_ < −2.46; 2.13 < H_P_ < 5.12) indicating that misclassifications were very unlikely.

Individuals were first acclimated to laboratory conditions for three days and kept in indoor tanks, with their original plant hosts, at ambient temperature and with filtered running seawater and artificial aeration. At the start of the experiments, shrimp were transferred to rectangular plastic aquaria (30 × 20 × 10 cm) and maintained at nearly constant temperature (25 °C). In all experiments, the position of aquaria assigned to different experimental treatments was randomly chosen to avoid potential artefacts due to uncontrolled spatial variation of any physical variables within the laboratory room.

### Algal preference

General procedures followed standard protocols for multiple-choice tests (e.g. [55, 56]). Algae were supplied in equivalent quantities (20 ml) as single clumps anchored to opposite corners of the aquaria (*n* = 12 for each morph). Fifteen individuals (H_GB_, H_P,_ or ST) were added to the centre of each aquarium and, after 3 days, algae were carefully enclosed in dip nets and the number of living shrimp counted. As a response variable, we used the difference between the shrimp found at *Sargassum* and *Galaxaura*, divided by the total number of shrimp remaining alive at the end of the experiment, to account for mortality (2.8 shrimps ± 0.3). These preference indices were compared among morphs using a 1-way ANOVA. The Student-Newman-Keuls (SNK) procedure was used for a posteriori comparisons. Confidence intervals (95 %) were additionally calculated for each morph.

### Colour change and camouflage

Previous observations indicated that the capacity of colour change differs between shrimp morphs, with H individuals visually changing their body colour in few days when exposed to an unmatched algal habitat, and ST shrimp being unable to change their coloration in the same period [9]. In this study we restricted further and more detailed analyses of colour change to the H morph. We cannot discard long-term colour shifts in ST shrimp, but because transparent individuals are typically characterized by a much reduced number of colour cells and pigments along the body, as observed in the closely related species *Hippolyte varians* [57] and *Heptacarpus pictus* [58], their eventual reorganization would likely respond to a different physiological process [28], acting over longer time-scales (weeks or months [27, 28]).

Here, we conducted an experiment to quantify colour change and camouflage in the plastic morph (H), exposing individuals of varying coloration (greenish-brown and pink) to different algal habitats and artificial substrates. By doing this, we aimed to (i) test whether short-term colour changes are possible on these substrates, (ii) examine if the mechanisms controlling colour change in this species depend on visual information or diet by keeping individuals on either artificial or natural substrates, with food resources only available in the latter, and (iii) compare the efficiency of colour alteration to provide camouflage in morphs exposed to colour matched and unmatched backgrounds. Although we acknowledge that it would have been ideal to do so, colour metrics were not quantified before the trials because handling of these small and fragile shrimp could likely alter their behaviour and cause excessive mortality. We therefore used the final colour of shrimp kept against a matching background as their standard in nature. This assumption was tested by comparing hue values (see below) between experimental shrimp on matching backgrounds with shrimp freshly collected in the field (*n* = 10 for each morph); i.e. experimental H_GB_ on *Sargassum* vs. natural H_GB_, and experimental H_P_ on *Galaxaura* vs. natural H_P_.

#### Image analyses

We measured colour for individual algae and shrimp in all experimental treatments using digital image analyses, which provides a powerful and non-invasive approach to quantify animal coloration [59]. A Nikon Coolpix P5000 camera, coupled to a stereomicroscope and a constant white light source of 3200 K colour temperature, was used to obtain all images. Samples were photographed using manual white balancing and exposure settings to avoid colour saturation [59], followed by photographs of one standard grey card (Color Checker Passport, X-Rite), reflecting light equally at 35 % between 400 and 750 nm, using the same camera settings, as required by the sequential method of calibration [60]. Before obtaining colour data, each image was linearised to control for changes in light intensity using a set of six grey references from the colour checker chart (Color Checker Passport, X-Rite), based on the methods described by Westland and Ripamonti [61] and Stevens et al. [59]. This procedure was necessary because many digital cameras show non-linear responses of image values to changes in light levels that need to be corrected before obtaining accurate data. The camera responses were also equalised in relation to the 35 % standard grey card to control for changes in the illuminating light conditions. Finally, images were scaled to reflectance values in red (longwave; LW), green (mediumwave; MW), and blue (shortwave; SW) layers (an image value of 255 on an 8-bit scale is equal to 100 % reflectance [59]).

For each shrimp or algal image, we measured regions of interest (ROIs) and sampled the values of reflectance in the red, green, and blue channels (RGB) using the program ImageJ [62]. For shrimp images, we selected one square (1.5 mm^2^) on the abdominal region of individuals, between somites 2 and 3, where colour is particularly uniform, and for algal images we selected the entire frond outline (approx. 50 mm^2^). For shrimp data, we obtained values of colour (hue), which was calculated as the red/green ratio, broadly analogous to the general principle of an opponent colour channels, whereby colour types are encoded by antagonistic neural pathways [63, 64] and similar to other past studies [37, 65]. Red, grey, and green tones would provide hue values > 1.0, ≈ 1.0 and < 1.0, respectively. The use of this metric does not depend of any specific visual system or predator group [64], allowing us to analyse colour in terms of the physical properties of each shrimp in an intuitive way.

#### Colour change

We prepared two replicate aquaria for each treatment combination of ‘morph’ (H_GB_, H_P_) and ‘background colour’ (brown, pink). Parallel trials were run using 20 ml substrates of either natural (brown *Sargassum* and pink *Galaxaura*) or artificial background (assembled stripes of brown and pink plastic tape), summing up 16 experimental units. Artificial substrates matched algal tones as closely as possible, while providing intermediate habitat architecture between the highly intricate *Sargassum* matrix and the smoother *Galaxaura* habitat. Seven to eight shrimp were initially added to each of these aquaria, with individuals maintained in artificial substrates supplied pellet shrimp food daily. Air pumps ensured adequate water circulation and aeration. In all treatments, individuals were recovered after 5 days, immediately frozen (a procedure that did not alter their colour), and later photographed to obtain colour values. A few shrimp were lost (possibly owing to mortality) and we had to reduce sample size to the minimum number of individuals found across aquaria (*n* = 5, for both parallel trials using natural and artificial substrates), ensuring a balanced design. Excess individuals from remaining aquaria were randomly excluded from analyses. To test the ability of individuals to change colour, we compared hue values separately for each experiment (natural or artificial substrates) using a mixed three-factor ANOVA in which factors ‘morph’ (H_GB_ or H_P_) and ‘substrate colour’ (brown or pink) were fixed and orthogonal, and the factor ‘aquaria’, with two levels, was random and nested in the interaction between main factors. The Student-Newman-Keuls (SNK) procedure was used for a posteriori comparisons.

#### Camouflage

We also aimed to quantify the efficiency of colour change to provide camouflage against both algae. For that, we compared the final colour of shrimp reared in the different experimental treatments to the actual colour of both *Sargassum* and *Galaxaura*. We first standardised the reflectance data in the three colour channels (RGB) of shrimp and algae and then converted these values to x and y coordinates in a trichromatic colour space [66]. Colour departures were calculated as the Euclidian distances between coordinates of replicate shrimp and algae. Replicate algal coordinates (*n* = 20) were randomly split in two groups, to provide independent and balanced distance estimates between algae and shrimp for each morph. We used *t*-tests, corrected for heteroscedasticity when needed, to compare colour coordinates of each shrimp morph against the colour of both algae, predicting that shrimp colour would be closer to the colour of their rearing background than to the colour of the alternative algal background.

### Morphological and behavioural differences between morphs

Intraspecific plasticity of body shape, which substantially affects hydrodynamics, is commonplace in a variety of aquatic invertebrates and fish, and may indicate differential patterns of habitat use and behaviour [15, 52, 53, 67]. Because H and ST morphs were differently distributed between algal habitats and possibly subjected to distinct selective forces [9], we predict that *H. obliquimanus* individuals will exhibit morph-specific shape, with possible consequences on shrimp behaviour and life-style. Since homogeneous individuals can change their colour in just a few days (see Results), we pooled the H_GB_ and H_P_ categories together in a single group (H) for follow-up comparisons on morphology and behaviour.

#### Morph-specific shape

We used geometric morphometric analyses to compare carapace shape differences between morphs. Analyses were restricted to males to eliminate any variability owing to sexual dimorphism. Twenty-one H and 25 ST individuals were sorted from samples of *Sargassum* and *Galaxaura* collected in the São Sebastião Channel (as in [9]). Shrimp were fixed in 70 % ethanol, stained with rose bengal, and their left carapace side was photographed using a Nikon Coolpix P5000 camera, coupled to a stereomicroscope set at a magnification power of 10×.

Nine landmarks were defined along the margin of the carapace as follows; 1: eye orbit, 2: rostral tip, 3: first dorsal spine, 4: mid-dorsal margin, vertically opposed to landmark 8, 5: posterior dorsal edge, 6: posterior lateral tip, 7: distal ventral margin, vertically opposed to landmark 5, 8: ventral-most point, opposite to landmark 4, 9: ventral insertion point of the antennule. Landmarks were defined using the software tpsDig 2.14 [68], following standardized criteria [69]. Landmark alignment and the acquisition of shape variables, both uniform components (UCs) and relative-warps (RWs), were carried out following the procedures described by Zelditch and co-workers [69], using the software tpsRelw 1.46 [70].

The values of UCs and RWs were separately compared between H and ST individuals, using multivariate analysis of variance (MANOVA). Centroid size (CS), i.e. the square root of the summed squared distances between all landmarks and the carapace centre of gravity (centroid), was used as a size variable and compared between colour morphs with a *t*-test.

#### Habitat fidelity and mobility

We compared substrate fidelity and individual mobility between morphs in a simple laboratory experiment. Trials were performed in plastic rectangular aquaria (30 × 20 × 10 cm) provided with a longitudinal flow of 2 l/min containing a single *Sargassum* clump (40 ml) attached to the upstream end, and 20 shrimps, 10 H_GB_ and 10 ST, at the opposite downstream side. We used only *Sargassum* as habitat in this experiment because this is the algal type supporting the highest shrimp density in the study area [9], and also because this is the preferred habitat of these colour morphs (see Results). The same experimental setup was replicated five times and, in each trial, all individuals were morph identified (Additional file 1: Figure S1) and monitored using a video camera (Sony HDR-XR250) for 30 min. Five three-minute video samples were selected for analyses, starting at time 1.5 min and taken at every other 3 min intervals, thus providing samples centred at times 3, 9, 15, 21 and 27 min. For each video sample we separated 90 frames (one every 2 s) for analyses. Habitat fidelity was estimated as the percentage of shrimp on algae at frame 45 (at the mid of each sample). In order to quantify mobility, we tracked the position of each shrimp remaining out of algae through all the 90 frames for each period and calculated total travelled distances. These analyses were undertaken using the software ImageJ.

The proportion of shrimp settled on algae was used as a proxy of shrimp habitat fidelity. Between-morph comparisons of these proportions, at different times, were examined using repeated-measures ANOVA because data from the same aquaria are dependent on time. Raw data were used since the sphericity assumption was met (*W* = 0.089; *p* = 0.078). Mobility of individuals was first estimated by comparing individual travelled distances between H and ST shrimp using Mann-Whitney tests. Comparisons on ranks did not detect differences between morphs (69.5 < *U* < 396.5, *p* > 0.05 for all sampled periods) because most individuals (72 %) moved very little around their initial positions, typically less than 2 cm. Therefore, we proceeded by comparing mobility of the fewer remaining shrimp that did swim considerable distances. Since these were outliers within the whole population (based on an outlier coefficient, *k*, of 2.0), we first subtracted swimming distances by baseline movement at their respective sampling period, i.e. the upper fence for non-outlying data. These corrected swimming distances were considered independent records and compared between morphs using a *t*-test.

## Results

### Algal preference

**Fig. 2:**
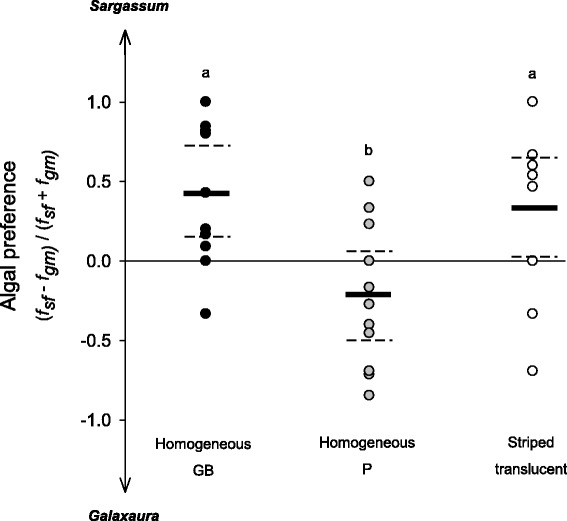
Algal preference of homogeneous (greenish-brown; GB and pink; P) and striped translucent colour morphs. f_sf_ and f_gm_ stand for the frequencies of shrimp occupying *Sargassum furcatum* and *Galaxaura marginata* fronds at the end of trials. *Solid* and *dashed black lines* denote mean values and ± 1 CI (95 %) respectively. *Different letters* indicate statistical differences among morphs (*p* < 0.05)

Shrimp colour morphs exhibited different preferences for algal substrates (ANOVA: *F*_(2,33)_ = 6.84, *p* = 0.003). When equal volumes of the two algal types were made simultaneously available to shrimp, both H_GB_ and ST morphs showed higher preference towards *Sargassum*, compared to H_P_ individuals (SNK tests, *p* < 0.01). Confidence intervals (95 %) indicate net preference for the brown weed for H_GB_ and ST but not for H_P_ shrimp (Fig. 2).

### Colour change and camouflage

**Fig. 3:**
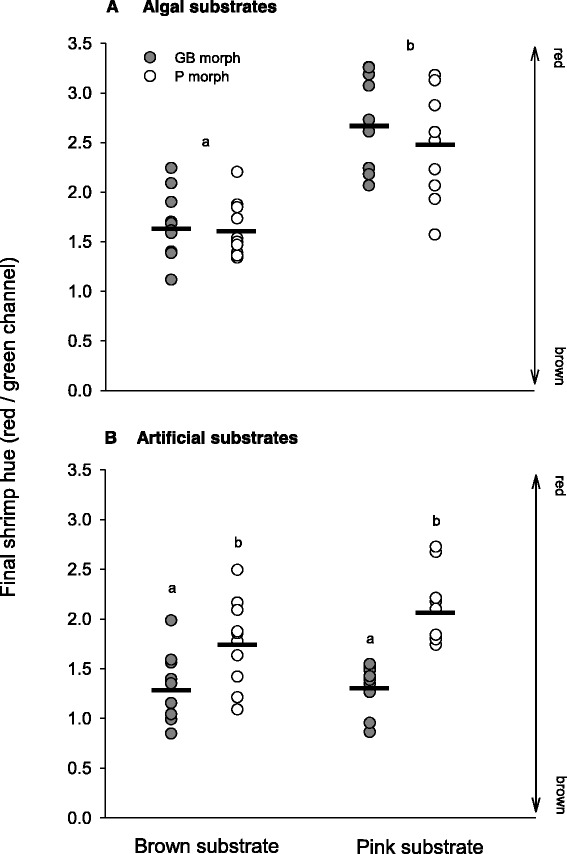
Colour change in homogeneous greenish‐brown (*GB*) and pink (*P*) individuals when exposed to **a** algal and **b** artificial substrates of brown and pink-red coloration for five days. Data from different aquaria, in each combination of ‘morph’ and ‘substrate colour’, were pooled. Final shrimp colour (hue) was defined as the ratio between reflectance in the red and green colour channels. Higher hue values correspond to reddish tones. Mean values are denoted by *solid black lines* and *different letters* indicate significant statistical differences between groups (*p* < 0.05)

Hue values of shrimp held on matching backgrounds are regarded as natural standards, since they did not significantly differ from hue values of respective counterparts in the field (experimental H_GB_ in *Sargassum* vs. natural H_GB_: *t*_18_ = 0.52, *p* = 0.609; experimental H_P_ in *Galaxaura* vs. natural H_P_: *t*_18_ = 0.86, *p* = 0.401). It is thus concluded that homogeneous shrimp (H_GB_ and H_P_) exposed to unmatched algal habitats were capable of pronounced colour change over the 5-day periods during which trials were undertaken (Table 1, Fig. 3a).

**Table 1:** Summary results of the mixed three-way analyses of variance testing the effects of morph type (M; greenish-brown or pink), substrate colour (S_C_; brown or pink) and aquaria (nested in the interaction between main factors) in final hue values measured in *Hippolyte obliquimanus* individuals after being maintained for five days in artificial or algal substrates

		Algae			Artificial substrates		
Source of variation	*df*	*MS*	*F*	*P*	*MS*	*F*	*p*
Morph (M)	1	0.170	0.92	0.392	3.750	44.13	0.002
Substrate colour (S_C_)	1	8.636	46.56	0.002	0.298	3.51	0.134
M x S_C_	1	0.069	0.37	0.574	0.217	2.55	0.185
Aquaria (M x S_C_)	4	0.186	1.07	0.388	0.085	0.65	0.632
Error	32	0.174			0.131		
		C = 0.247; ns			C = 0.240; ns		

*C* Cochran statistic, *ns* not-significant

Colour change was very clear in natural algal substrates but not in artificial ones. H_GB_ individuals increased their hue values after being in contact with the red alga *Galaxaura*, attaining a reddish coloration, and H_P_ shrimp showed the opposite pattern when placed in *Sargassum*, achieving at the end of the experiment a brownish tone (Fig. 3a). As a result, hue differences between shrimp morphs, within each algal habitat, disappeared at the end of the trial (Fig. 3a; SNK tests, *p* > 0.05). However, shrimp reared in artificial substrates retained morph-specific hue (thus the significance of ‘morph’, Table 1), with no changes toward background colour (Table 1; Fig. 3b). Hue differences between morphs persisted both in brown (SNK test, *p* < 0.05) and pink (SNK test, *p* < 0.01) artificial substrates (Table 1).

**Fig. 4:**
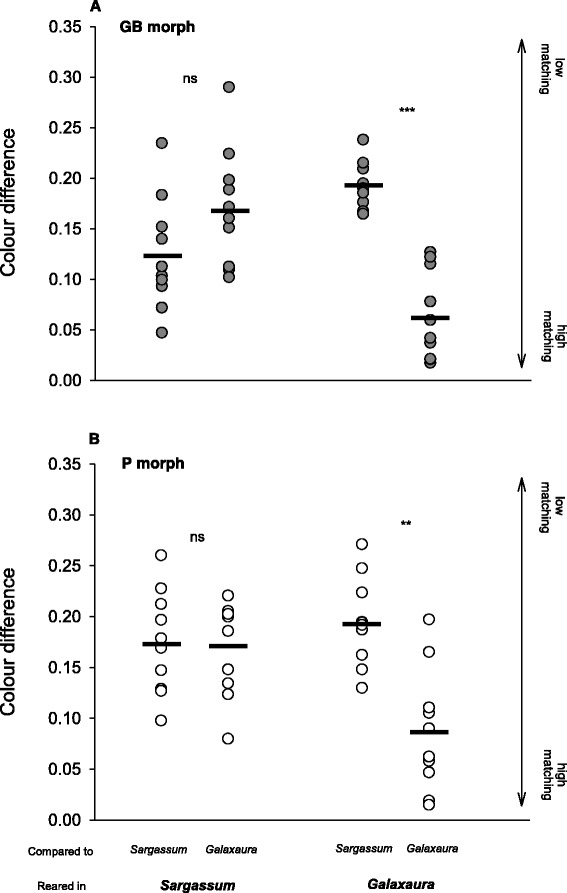
Colour differences between **a** homogeneous greenish‐brown (*GB*) and **b** homogeneous pink (*P*) shrimp and the algae *Sargassum* and *Galaxaura*, for groups of individuals reared for five days in each of these substrates. Colour differences were defined as the Euclidian distance between standardised colour coordinates in a trichromatic reflectance colour space of shrimp and algae. *Solid black lines* represent mean group values. **p < 0.01; ***p < 0.001; *ns*: not-significant

Although both shrimp morphs were capable of changing colour when exposed to unmatched natural backgrounds, the effectiveness of this change in promoting camouflage depended on the algal type shrimp were been placed on. Overall results suggest that colour camouflage is more efficient in the pink alga *Galaxaura*. H_GB_ shrimp reared in *Sargassum* ended up with a coloration equally distant from *Sargassum* and *Galaxaura* (Fig. 4a; two sample *t*-test: *t*_*(*18)_ = 1.85, *p* = 0.080). However, when H_GB_ individuals were placed in the unmatched *Galaxaura* background they were capable of changing their colour remarkably well, becoming quite close to *Galaxaura*, and very different from the *Sargassum* background (Fig. 4a; two sample *t*-test: *t*_*(*18)_ = 7.16, *p* < 0.001). Very similar outcomes were observed for H_P_ shrimp. When individuals were placed on *Sargassum*, they did change colour but ended with values equidistant from both algal types (Fig. 4b; two sample *t*-test: *t*_*(*18)_ = 0.91, *p* = 0. 114), indicating poor camouflage. When reared on the matching substrate *Galaxaura*, the final colour of individuals was again very similar to *Galaxaura* but distant to *Sargassum* (Fig. 4b; two sample *t*-test: *t*_*(*18)_ = 4.63, *p* < 0.001).

### Morph-specific morphological and behavioural patterns

#### Morph-specific shape

**Fig. 5:**
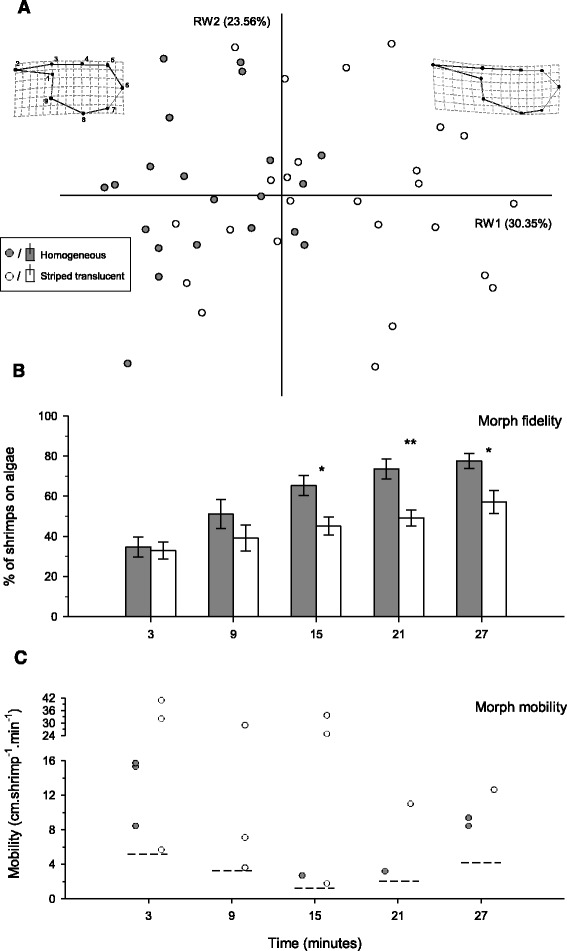
Proxy variables indicating different life-styles in homogeneous (H; in *grey circles or bars*) and striped translucent morphs (ST; in *white circles or bars*). **a** Carapace shape differences based on geometric morphometric results. Morphotypes are clearly segregated along the first relative warp axis, from a stout carapace outline representative of H individuals, to a more streamlined shape found in ST shrimp. Percentage values represent the relative warps share of the total morphological variation. Dots along carapace margins show the position of landmarks used in the analysis (see Methods). **b** Substrate fidelity of morphs, over 30 min experimental trials, expressed as the percentage of individuals settled on algal clumps. Whiskers represent ± 1 SE. *p < 0.05; **p < 0.01. **c** Morph-specific mobility, expressed as individual average swimming distances (in cm) per minute, over 30 min experimental trials. Measurements for mobile shrimp are outliers (*filled circles*) from baseline movement of sedentary individuals. *Dashed lines* represent the upper fence delimiting the non-outlier range (see Methods for details)

Centroid size did not vary between homogeneous and striped translucent males (two sample *t*-test: *t*_(44)_ = 1.10, *p* = 0.277), i.e. H and ST shrimp were of similar size. However, shape differences were clear. Fourteen shape variables (relative warps – RWs; i.e. axes showing major trends of localised shape variation [69]) were obtained, with the three most important ones explaining 63 % of the whole overall shape variation. MANOVA results, applied to all relative warps axes, indicated shape contrasts between colour morphs (MANOVA Wilks test: *F*_(14,31)_ = 3.60, *p* = 0.001). Of greatest importance was RW1, accounting for the greatest percentage variance (30.4 %) and clearly segregating morphs. H shrimp were mostly distributed along the negative side of RW1, which corresponds to a stouter carapace shape, while ST individuals were mostly distributed along the positive side of the axis, corresponding to a streamlined carapace shape (Fig. 5a). A MANOVA analysis applied to the two uniform components (UCs) further suggested a difference between colour morphs (MANOVA Wilks test: *F*_(2,43)_ = 4.82, *p* = 0.013). As RW1, UC1 explained almost all morphological variation between morphs. Such a component refers to uniform contraction/expansion of the whole body, and segregated H shrimp at the negative axis half (carapace dorso-ventrally expanded), and ST individuals at the positive one (carapace dorso-ventrally compressed).

#### Habitat fidelity and mobility

Substrate fidelity was markedly different between H and ST shrimp over time (repeated-measures ANOVA: *F*_(4,32)_ = 2.77, *p* = 0.044; Fig. 5b). At the beginning of the experiment (3 min), the proportion of individuals found on algal clumps was low, but virtually the same for each morph. The number of shrimp using the algal habitat tended to increase through time, but the rate at which they stopped swimming and settled on algae differed between H and ST shrimp. At 9 min, differences were already noticeable, increasing thereafter to statistical significance. At the end of the experiment (27 min), 78 % of H shrimp but only 57 % of ST individuals had settled on algae (Fig. 5b).

Mobility above baseline activity was restricted for a small fraction of the population and decreased from 12 % to 6 % over the experiment (Fig. 5c). Most of these swimming individuals were ST shrimp (61 %). Considering all sampled periods, average mobility was higher in ST (15.2 cm.shrimp^−1^.min^−1^) than in H shrimp (5.20 cm.shrimp^−1^.min^−1^; two-sample *t*-test: *t*_(17)_ = 2.20, *p* = 0.043). It is also important to note that swimming events over distances larger than 25 cm each minute (*n* = 5) were only recorded for ST shrimp (Fig. 5c).

## Discussion

We report contrasting behavioural and morphological patterns in colour morphs of the shrimp *Hippolyte obliquimanus*, suggesting a diversification of life-styles between morphs which can be linked to alternative camouflage strategies. Our results indicate that H shrimp are capable of fast colour change, with different colour types concealed in distinct macroalgal habitats. Individuals of this morph are also tightly connected to their benthic habitat, avoiding long-distance swimming away from their host algae, which explains why they concentrate in exceptionally high densities in the brown weed *Sargassum* [9]. All these features suggest that this morph presents a specialist camouflage strategy, achieved by concealment to a specific background type (at any given point in time, although individuals can change colour over time). In contrast, ST shrimp cannot rapidly adjust their colour to their background environment, and also show low habitat fidelity and substantial swimming activity, indicating a more pelagic life-style. These characteristics are in accordance to their uniform distribution between *Sargassum* and *Galaxaura*, the two main vegetated habitats in the study region [9], suggesting a generalist habitat use linked to a camouflage strategy achieved by transparency. It is noteworthy that the results of experiments on behavioural patterns are consistent with morphological analyses, indicating a more benthic life-style for H shrimp and a more pelagic habit for ST shrimp, encompassing an important range of the morphological variation found in caridean shrimp [52, 53].

Colour change in H shrimp was observed upon contact with living algal habitats, but not artificial substrates, indicating the process of colour change in this species, and possibly in many other algal-dwelling isopods [71], decapods [7, 57] and fish [72], relies, at least in part, on substrate-individual interactions. In fact, some authors have shown that the ingestion of carotenoid pigments can promote colour change in other crustaceans [73, 74], typically over a longer period (weeks) than observed in this study. Note that this does not discount a role of visual feedback, and future work should independently change diet and visual appearance to tease apart these effects.

Colour change may be a faster process for small crustaceans shedding thin translucent exuviae (own observations) than large ones, because pigment reorganization in hypodermic colour cells may be readily visible, as observed for another hippolytid shrimp species [57]. Colour change in H shrimp strongly suggests a camouflage strategy by background matching, whereby individuals’ overall body colour, colour pattern, and brightness tend to resemble the general background [75]. However, we observed H shrimp concealed better on the pink *Galaxaura* than on the brown *Sargassum*. In *Sargassum*, H_GB_ and H_P_ ended up with an intermediate body colour pattern, equally distant from the two algal types. In contrast, both shrimp morphs reared in *Galaxaura* became much better concealed to this substrate than to the alternative *Sargassum* background. These results were surprising since natural shrimp densities in the brown *Sargassum* are far higher than in *Galaxaura* [9]; a difference that could be explained by more efficient camouflage in the former. Our results, however, indicate that this is not the case, and that factors other than colour camouflage alone likely underlie this species distribution in the field. Also, these findings are aligned to ongoing research suggesting better protection against predators in *Galaxaura* (in prep), highlighting the importance of concealment in the pink weed habitat. Further work on longer term changes in colour than those tested here are also needed.

The *Sargassum* and *Galaxaura* canopy constitute the most important habitat types to shrimp in our study area, but the relative value of these habitats for *H. obliquimanus* is apparently very different [9]. Experiments in the laboratory testing algal preferences showed that H_GB_ and ST individuals actively select *Sargassum* fronds while H_P_ shrimp did not show any significant preference, indicating that colour camouflage is not an important selective force setting patterns of habitat choices. Strong preference of individuals for *Sargassum* may be adaptive for several different reasons not addressed in this study. For instance, as a much more physically complex habitat, especially when associated to epiphytic algae (e.g. *Hypnea* spp. [76, 77]), *Sargassum* would probably supply better shelter from predators and more extensive foraging grounds [78, 79] compared to *Galaxaura*. It is also possible that inconspicuous behaviour coupled to shape resemblance to background details [18, 80, 81] in the more complex *Sargassum* would ultimately render superior predator avoidance. More specific research addressing these issues is pending.

Habitat fidelity and mobility further support morph-specific life-styles. Colour-changing shrimp (H) show higher substrate fidelity and lower mobility rates indicating a more specialized habitat use. Although capable of colour alteration towards background matching, moving from one algal habitat to another would likely come at a cost. Settling on non-matching habitat for even a few days, compatible to the time for colour adjustment, may lead to very high predation rates [82–84]. Colour change may also carry physiological costs, although these have rarely if ever been quantified [26]. Therefore, at any one time, H morphs may be able to conceal to a specific background type, being considered background specialists. Conversely, ST shrimp may be generally concealed against a wider range of visual backgrounds [85] while moving from one habitat patch to another. Therefore, the transparency of individuals, linked to a higher mobility and lack of substrate fidelity, may eventually promote camouflage by means of a strategy independent (or partially dependent) of background matching, indicating a more generalist type of concealment and habitat use [33, 35, 39]. Morph-specific life-styles are supported by natural shrimp distributions [9] and also by geometric morphometrics analyses of carapace shape. The morphological gradient observed overlaps a great deal of the variation for caridean shrimp in general [52, 53]. While the more hydrodynamic shape found in ST shrimp clearly resembles the shape of pelagic shrimp species, the stouter H morphology are more akin to benthic species. More streamlined ST shrimp swimming distances within the range of 25 to 45 cm each minute may easily move across different algal habitats, which is not the case of more sedentary and deep-bodied H shrimp that were never observed swimming over such distances and tended to settle and remain on algae more frequently. Shrimp morphology, perhaps coupled to behaviour, may also affect camouflage in their algal habitats. Further experimental work is required, however, to examine this issue more closely.

While the different colour types of H and ST individuals may reflect distinct life-styles, we might ask what drives selection for these different approaches. Low dispersal and optimization of resource use can be particularly advantageous in H individuals, which concentrate in habitat patches where shelter is abundant and/or camouflage efficient. Even being a habitat where colour camouflage does not appear to be critical, *Sargassum* supports high densities of H shrimp, which exhibit high preference and fidelity to this habitat. The less structured *Galaxaura* substrate would be important as a secondary habitat to this morph, where colour concealment will be a valuable mechanism to reduce prey detection by visual predators. Based on these assumptions, we may expect strong intraspecific competition in *Sargassum* habitat, and hence selection for optimal resource use and territorial behaviour, which would possibly lead to displacement of ST individuals to *Galaxaura*. Density-dependent processes and loss of preferred habitats could be major mechanisms regulating abundance of H individuals. On the other hand, high dispersal potential and a generalist habitat use may be useful traits for ST shrimp. Because ST shrimp are mainly males [9], intense mobility and low substrate fidelity would likely enable males to find more mates in a pure-search strategy, expected for polyginic caridean species such as *H. obliquimanus* [45, 86, 87].

The coexistence within a population of distinct morphs with alternative strategies of habitat use and camouflage, as observed for *H. obliquimanus*, facilitates diversification on the use of environmental resources [9] and can also have ecological and evolutionary consequences, mainly on population stability over time [5]. The availability of the presumably higher-quality *Sargassum* habitat in our study region is markedly seasonal, with very high cover during summer and a much reduced density in winter, sometimes collapsing in that season [88]. Temporal variation in *Sargassum* cover can be a major mechanism controlling H shrimp densities, once individuals show strong specialization for this habitat. Therefore, the existence of an alternative habitat (*Galaxaura*) and morphs differing in their degree of habitat specialization may allow temporal changes in individual fitness associated with habitat availability and morphs density and frequency. Ongoing research on trophic niche space would further elucidate morph-specific patterns of resource use.

## Conclusions

Colour camouflage is a common anti-predator strategy in nature, but few studies investigate complex interactions among colour traits and other morphological and behavioural mechanisms, indicative of general morph-specific life-styles. Our findings illustrate that specific arrangements among morphology, behaviour, and (micro-) habitat use in colour morphs of the algal-dwelling shrimp *H. obliquimanus* may result in a diversification of camouflage strategies in a species living in a heterogeneous habitat. Colour change ability and high substrate fidelity, associated to a more robust morphology, suggest a specialist camouflage strategy in H individuals. On the other hand, high mobility coupled with a more streamlined morphology and lack of substrate fidelity in ST individuals, indicate a general strategy of camouflage in this morph. Higher mobility of the ST morph, in which more than 70 % of individuals are males [9], may also sustain a pure-search polygynic mating strategy which is predicted for this species. Seasonal changes on macroalgal cover may affect the frequency and fitness of the different colour morphs in the population. Selective mechanisms, such as morph-specific predation by visual consumers through contrasting patterns of habitat use [51, 89], would be important forces maintaining the diversification of life-styles and camouflage strategies in this shrimp species.

## Additional files

Additional file 1: Figure S1. Images extracted from video footage showing the experimental set up used in habitat fidelity and mobility trials (see details in the main text). Homogeneous (H) and striped translucent (ST) morphs of the shrimp *Hippolyte obliquimanus* were more easily identified in dorsal view, when thin longitudinal stripes of ST shrimps stood clearly out from the bottom of aquaria, contrasting to the solid coloration typical of H shrimp. A lateral view of an ST individual (as the lowermost shrimp in the lower image), showing translucent areas over the abdomen and carapace, could however suffice for morph identification. (PDF 489 kb)
Additional file 2: Supporting data. (XLSX 39 kb)
