# Supplementary material for: Shape, colour plasticity, and habitat use indicate morph-specific camouflage strategies in a marine shrimp
Source: BMC Evol Biol. 2016 Oct 18;16:218. doi: 10.1186/s12862-016-0796-8 (PMC5070350; doi:10.1186/s12862-016-0796-8)
Supplement: Additional file 1: Figure S1. — Images extracted from video footage showing the experimental set up used in habitat fidelity and mobility trials (see details in the main text). Homogeneous (H) and striped translucent (ST) morphs of the shrimp Hippolyte obliquimanus were more easily identified in dorsal view, when thin longitudinal stripes of ST shrimps stood clearly out from the bottom of aquaria, contrasting to the solid coloration typical of H shrimp. A lateral view of an ST individual (as the lowermost shrimp in the lower image), showing translucent areas over the abdomen and carapace, could however suffice for morph identification. (PDF 489 kb) [file 12862_2016_796_MOESM1_ESM.pdf]

Online Supplementary Material

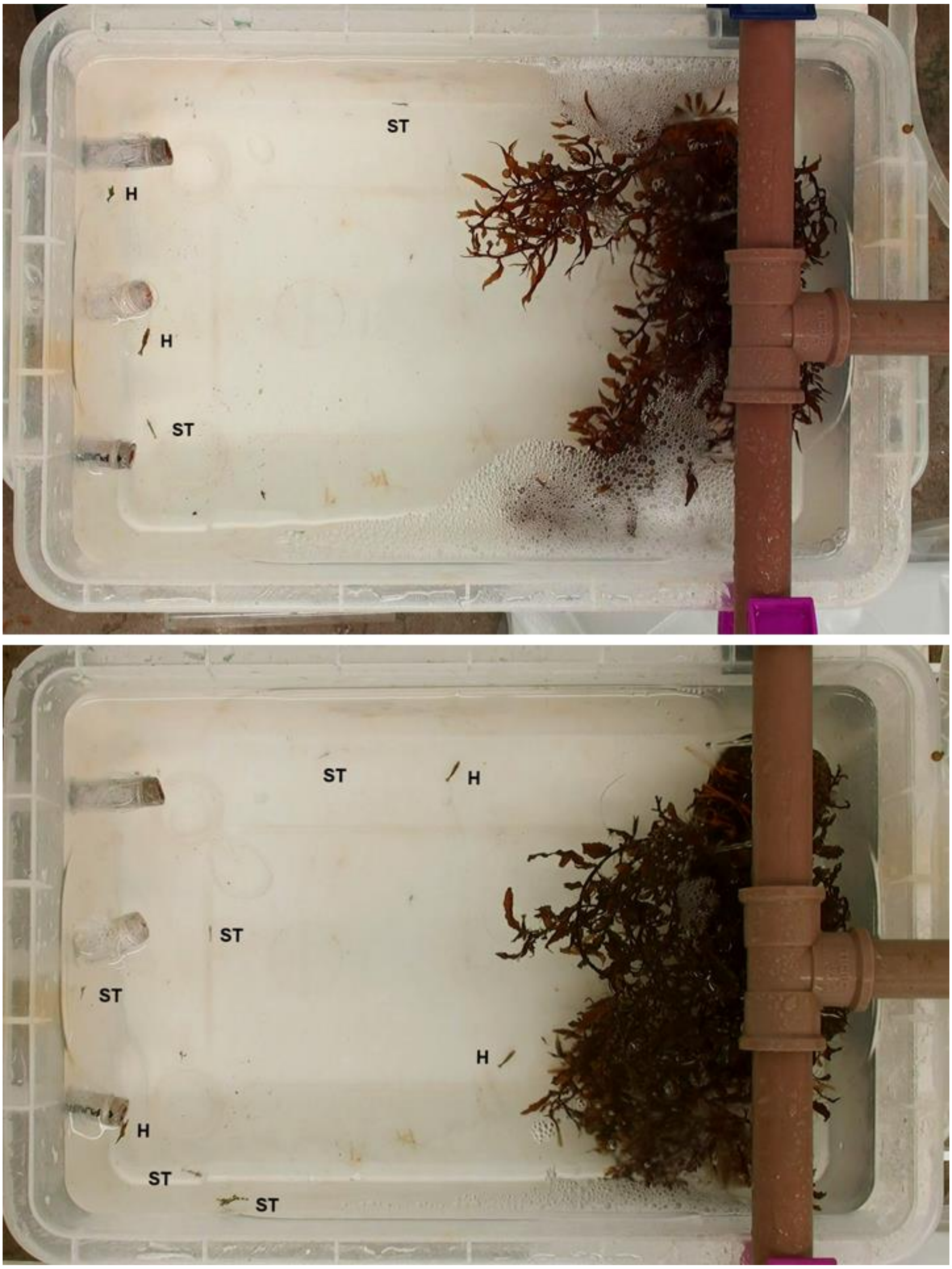

Fig S1 Images extracted from video footage showing the experimental set up used in habitat fidelity and mobility trials (see details in the main text). Homogeneous (H) and striped translucent (ST) morphs of the shrimp *Hippolyte obliquimanus* were more easily identified in dorsal view, when thin longitudinal stripes of ST shrimp stood clearly out from the bottom of aquaria, contrasting to the solid coloration typical of H shrimp. A lateral view of an ST individual (as the lowermost shrimp in the

lower image), showing translucent areas over the abdomen and carapace, could however suffice for morph identification.
